# Visualization of injectate spread of intercostal nerve block: a cadaveric study

**DOI:** 10.1186/s40981-018-0204-z

**Published:** 2018-09-06

**Authors:** Yuichi Ohgoshi, Yosuke Usui, Satoshi Terada, Yoshimasa Takeda, Aiji Ohtsuka, Kenjiro Matsuno, Yasuhisa Okuda

**Affiliations:** 1 Department of Anesthesiology, Keiyu Hospital, 3-7-3 Minatomirai, Nishi-ku, Yokohama, Kanagawa 220-8521 Japan; 2 Department of Anatomy (Macro), Dokkyo Medical University School of Medicine, Mibu, Tochigi Japan; 3 Mizutani Pain Clinic, Shizuka, Japan; 4 Department of Anesthesiology, Dokkyo Medical University Saitama Medical Center, Koshigaya, Japan; 5 Department of Anesthesiology, Okayama University Medical School, Okayama, Japan; 6 Department of Human Morphology, Okayama University Graduate School of Medicine, Dentistry and Pharmaceutical Sciences, Okayama, Japan

**Keywords:** Intercostal nerve block, Neurolysis, Paravertebral block, Total spinal anesthesia, Paraplegia, Cadaver

## Abstract

**Introduction:**

Intercostal nerve block and neurolysis are widely used procedures, but their injectate spread has not been well understood. Previous studies have reported unexpected outcomes (paravertebral or epidural anesthesia) and spinal cord injury after intercostal nerve block and neurolysis. To investigate a possible mechanism for these complications, we aimed to visualize the flow of liquid injected near the intercostal nerve, using cadavers.

**Methods:**

We performed a simulated intercostal nerve block study using two Thiel-embalmed cadavers. Dye was injected into the interfascial plane between the internal and innermost intercostal muscles under ultrasound guidance (blue, 10 ml) or under direct vision (green, 5 ml).

**Results:**

Dye leakage began with injection of only 0.5–2 ml and occurred between the innermost intercostal muscle fibers. The dye injected around the intercostal nerve penetrated into the extrapleural space and reached the paravertebral space.

**Conclusions:**

Injectate placed around the intercostal nerve easily penetrate the extrapleural space and reach the paravertebral space. Intercostal nerve block or neurolysis has a risk of impairing at least the sympathetic chain and conceivably affecting the central nervous system.

## Background

Intercostal nerve block is one of the most widely used procedures for the treatment of acute or chronic chest wall pain. However, injectate spread from the intercostal nerve block has not been well understood. In the 1980s, a few reports suggested the possibility that injectate placed to establish an intercostal nerve block spread into the paravertebral space [1–3] or the epidural space [4]. Moreover, previous reports have found that total spinal anesthesia [5, 6] and persistent paraplegia [7–10] could occur after intercostal nerve block and neurolysis, respectively. The mechanisms by which local anesthetic and neurolytic agents, including phenol and alcohol, injected around the intercostal nerve (1) reach the paravertebral space or epidural space and (2) cause complications in the spinal cord remain unclear. To investigate a possible mechanism of these phenomena, we aimed to visualize the dynamic spreading of injectate following intercostal nerve block and neurolysis, using cadavers.

## Methods

Ethical approval for this study was provided by the Institutional Ethics Committee of Dokkyo Medical University (approval number ST-2901). The simulated intercostal nerve block study was performed using two Thiel-embalmed cadavers. Before dye injection, we removed the anterior portion of the thorax and enucleated the lung and the heart. The descending aorta and the vena cava were not excised, to avoid unintentionally injuring the parietal pleura and the paravertebral space. This study comprised the following two phases.

### Study 1: ultrasound-guided injection from outside of the thorax

The cadavers were placed in the lateral decubitus position. A linear transducer was placed in a longitudinal orientation 10 cm lateral to the posterior median line, and a 22-gauge needle (22-G, 0.7 mm × 80 mm, Plexufix, B-BRAUN, Tokyo, Japan) was advanced into the interfascial plane between the internal and innermost intercostal muscles. Green dye (water-based acrylic dye, 10 ml) was gently injected, and the spread of the dye was observed from the anterior side.

### Study 2: direct injection from the inside of the thorax

The cadavers were placed in the supine position. After excision of the parietal pleura, a small incision was made in the innermost intercostal muscle to expose the intercostal nerve. A 22-gauge needle was bent into a thoracic curve and inserted along the intercostal nerve into the interfascial plane between the internal and innermost intercostal muscles. The needle tip was located on approximately the medial axillary line. The needle entry point was occluded by a finger to prevent dye leakage, and 5 ml of blue dye was injected.

## Results and discussion

### Simulated intercostal nerve block

#### Study 1: ultrasound-guided injection from outside of the thorax

**Fig. 1:**
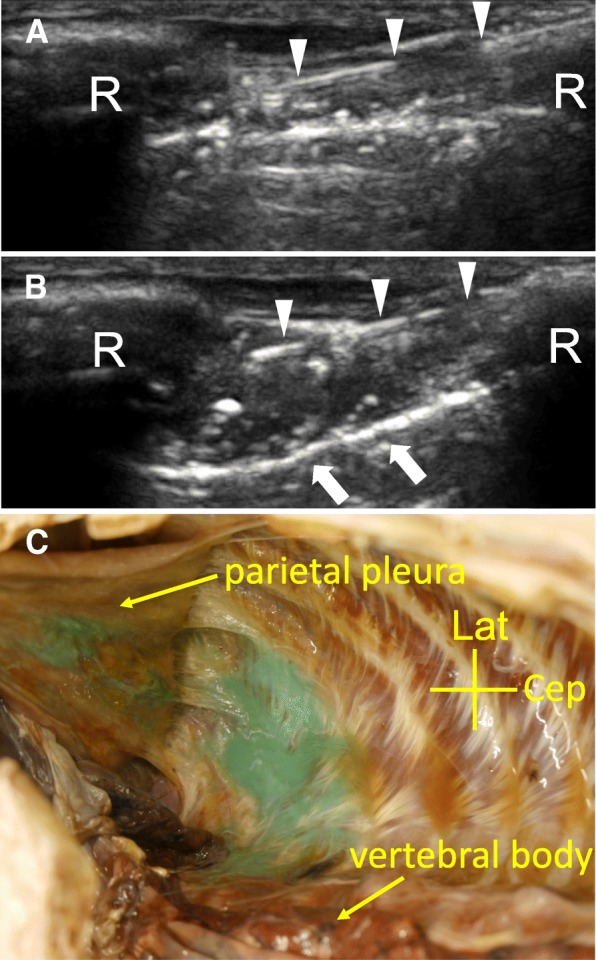
Ultrasound-guided dye injection from outside of the thorax. **a**, **b** Ultrasound images of the intercostal space before (**a**) and during (**b**) dye injection around the intercostal nerve in a cadaver. Arrow-heads show the needle, and arrows show the descent of the parietal pleura R rib. **c** Photograph showing the spread of dye from the extrapleural to the paravertebral space in a cadaver. The parietal pleura is turned outwards. Cep cephalad, Lat lateral

Dye injection from outside the thorax was performed in two cadavers. Figure 1a, b shows ultrasound images of dye injection between the internal and innermost intercostal muscles. Depression of the parietal pleura and dye leakage into the extrapleural space were observed immediately after injecting the dye solution in both specimens. After resection of the parietal pleura, the green dye was present in the parietal pleura, extrapleural space, and paravertebral space (Fig. 1c).

#### Study 2: direct injection from the inside of the thorax

**Fig. 2:**
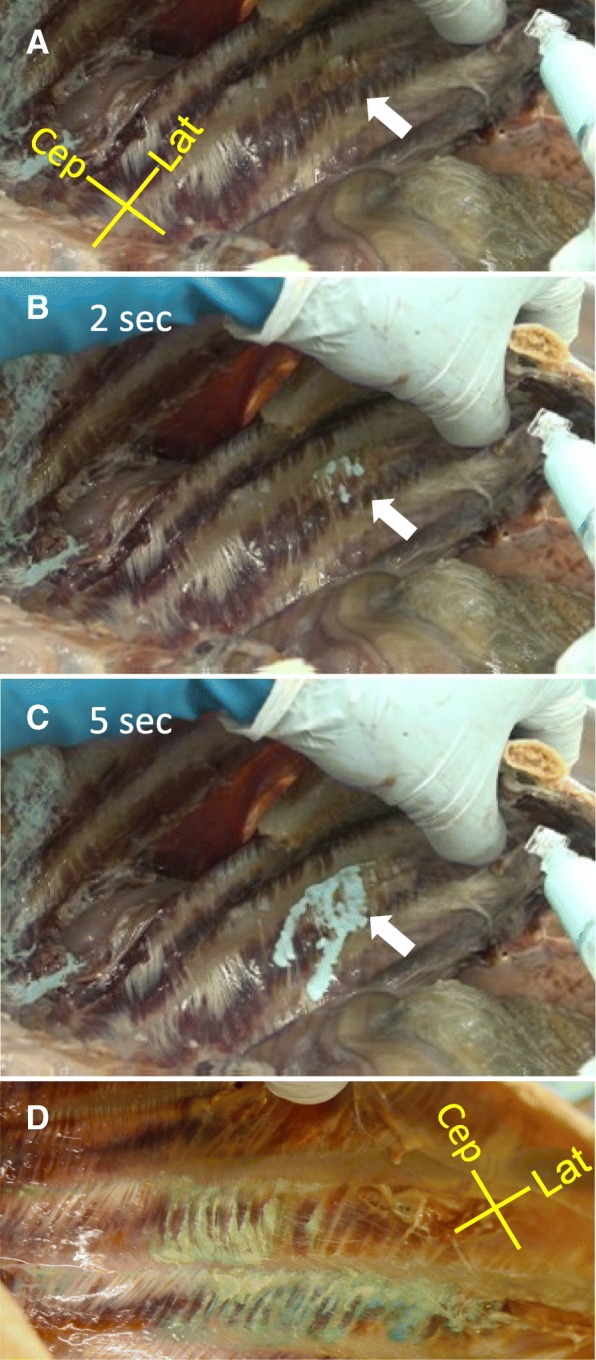
Direct injection of dye from the inside of the thorax. **a**–**c** Photographs showing the time course of dye spreading during injection into the interfascial plane between the internal and innermost intercostal muscles in a cadaver. White arrows show the dye injection point. Cep cephalad, Lat lateral. **d** Photograph showing other intercostal segments after dye injection. Dye deposition was observed between the muscle fibers

Dye injection from the inside of the thorax was performed in another 16 intercostal segments in two cadavers. For the 16 injections, dye leakage from the innermost intercostal muscle was observed in 14 segments (Fig. 2a–c). Dye leakage began with injection of only 0.5–2 ml and occurred between muscle fibers (Fig. 2d). There were two segments in which dye leakage was not observed; for these, subsequent dissection revealed that the needle had been misdirected into the incorrect plane, between the internal and external intercostal muscles, and that the intercostal nerves in these two segments were not stained.

In this visualization study, we aimed to clarify the movement of liquid that was injected to establish an intercostal nerve block or neurolysis, using cadavers. Our cadaver experiments provide two important findings. First, even a small amount of liquid placed near the intercostal nerve can penetrate through the innermost intercostal muscle into the extrapleural space. Second, liquid penetrating the extrapleural space can diffuse with gravity into the paravertebral space. Therefore, it is possible that these three compartments (the paravertebral space, the extrapleural space, and the intercostal nerve plane) can function as a connected series of spaces. To the best of our knowledge, previous studies using X-ray, computed tomography imaging, and cadavers have shown that agents injected around the intercostal nerve can reach the paravertebral space directly [1, 11]. In this study, we reported for the first time that agents injected to establish intercostal nerve block/neurolysis may also reach the paravertebral space via the extrapleural space.

Previous studies have reported that total spinal anesthesia [5, 6] and persistent paraplegia [7–10] occurred after intercostal nerve block and neurolysis, respectively. The mechanisms of these significant complications have been unclear. Our findings may shed some light on their true nature. The current study shows visually that agents injected around the intercostal nerve easily penetrate the extrapleural space and spread into the paravertebral space. Although migration of the liquid from the paravertebral space to the extradural space or the subarachnoid space does not frequently occur, it is not surprising that this can happen, depending upon the patient’s condition (e.g., body position, trivial trauma of the dura mater and the arachnoid, disc herniation, or vertebral metastasis of cancer). This assumption is supported by a previous cadaveric study showing that dye injected into the paravertebral space can spread at least to the extradural space [11]. Moreover, a case report has been published in which the use of a large volume of local anesthetic in intercostal nerve block resulted in epidural blockage [4]. Agents injected around the intercostal nerve can reach the paravertebral space and the sympathetic chain and easily impair at least the sympathetic chain.

On the other hand, the current study also shows that liquid penetration from the intercostal nerve plane into the extrapleural space occurred with only 0.5–2 ml of dye injection. Previous case reports of persistent paraplegia described the use of 2–6.7 ml of agents per segment (total volume 2–20 ml); this volume is large enough to flow into the paravertebral space. Our findings indicate that intercostal nerve block or neurolysis lead to paravertebral anesthesia and, conceivably, to subsequent spinal cord injury more easily than previously believed; thus, we must take special care when performing these procedures.

The limitations of this study include the use of embalmed cadavers, as embalmed tissue may not be representative of normal tissue integrity and is likely to influence the way in which aqueous fluids pass through the extrapleural space. Furthermore, the body position and existence or nonexistence of respiratory excursion are factors that may affect extrapleural fluid spread. We used water-based acrylic dye as a substitute for local anesthetic and phenol solution in each study. The chemical nature of the injectate is also a factor that may affect the outcome. We used two different volumes in each study. Although we chose 10 ml of dye to clearly demonstrate liquid spread in study 1, we chose 5 ml of dye for study 2 to demonstrate liquid penetration using multiple intercostal segments.

## Conclusions

Injectate placed around the intercostal nerve in cadavers easily penetrates the extrapleural space and reaches the paravertebral space. Intercostal nerve block and neurolysis can easily impair at least the sympathetic chain; thus, we must take special care when performing these procedures.
